# Effect of Two Combined Functional Additives on Yoghurt Properties

**DOI:** 10.3390/foods10061159

**Published:** 2021-05-21

**Authors:** Marek Szołtysik, Alicja Z. Kucharska, Anna Dąbrowska, Tomasz Zięba, Łukasz Bobak, Józefa Chrzanowska

**Affiliations:** 1 Department of Functional Food Products Development, Wrocław University of Environmental and Life Sciences, Chełmońskiego Str. 37, 51-640 Wrocław, Poland; anna.dabrowska@upwr.edu.pl (A.D.); lukasz.bobak@upwr.edu.pl (Ł.B.); jozefa.chrzanowska@upwr.edu.pl (J.C.); 2 Department of Fruit, Vegetable and Plant Nutraceutical Technology, Wrocław University of Environmental and Life Sciences, Chełmońskiego Str. 37, 51-640 Wrocław, Poland; alicja.kucharska@upwr.edu.pl; 3 Department of Food Storage and Technology, Wrocław University of Environmental and Life Sciences, Chełmońskiego Str. 37, 51-640 Wrocław, Poland; tomasz.zieba@upwr.edu.pl

**Keywords:** honeysuckle berries extract, resistant starch, yoghurt, antioxidative potential

## Abstract

The aim of the research was the analysis of yoghurts enriched with blue honeysuckle berries dry polyphenolic extract and new preparation of resistant starch. The additives were introduced individually at concentration 0.1% (*w/v*) and in mixture at final concentration of 0.1 and 0.2% of both components. Yogurt microflora, pH, and its physicochemical and antioxidant properties were examined over 14 days of storage under refrigerated conditions. Studies showed that both substances can be successfully used in yoghurt production. Yoghurt microflora es. *S. thermophilus* and *Lb. delbrueckii* subsp. *bulgaricus* counts appeared to be higher in samples supplemented with these additives comparing to control yoghurt by 3–8%. More stimulating effect on their growth, especially on *S. thermophilus*, revealed resistant starch. Addition of this polysaccharide improved also the rheological properties of yogurts, which showed higher viscosity than samples produced without it. Addition of honeysuckle berries preparation significantly influenced the yogurts’ color, giving them deep purple color, and their antioxidant potential. During storage, contents of anthocyanin and iridoid compounds were decreasing, but antioxidant activity in the products remained stable.

## 1. Introduction

In recent years, an increased interest in food products with positive effect on health beyond their nutritional value has been observed. Among such food products, much attention has been focused on fermented milk drinks, like yoghurt. Their beneficial properties are attributed to increased protein digestibility, reduced lactose content, antimicrobial activity, and immune system stimulating effect. These products are often enriched with different functional substances for improvement of their rheological or organoleptic properties as well as health promoting effects [1]. Among these substances, especially attractive is a variety of hydrocolloids and, originating from plants, different bioactive compounds including phenols, carotenoids, lycopene or essential oils, which reveal high antioxidant activity. Exceptionally rich sources of these latter compounds are, belonging to different genera, dark colored berries, such as bilberry, blueberry, chokeberry, black currant, or elderberry [2,3,4,5,6]. Some of them are widely used in yoghurt production in the form of fresh or processed fruits (pulps, juices) [7,8,9,10,11]. Their addition results in an improvement in nutritional and sensory properties of yoghurt. However, stability of such products during storage is not high due to color changes resulted from degradation of polyphenolic compounds or contamination with yeast and molds originating often from fruit additives [7]. Content and composition of bioactive compounds in plants (fruits and vegetables) is significantly influenced by species, cultivar, genotype, environmental conditions, maturity stage and time of harvest, and the subsequent storage conditions [12,13,14,15].

Lately, honeysuckle berries (*Lonicera caerulea L. var. kamtschatica*) have gained a lot of popularity in Poland and in other countries in Europe and North America. This plant species belongs the *Lonicera* genus of *Caprifoliaceae* family and is native to Siberia, North Eastern Asia, and Japan. It is also named blue honeysuckle, honeyberry, edible honeysuckle, or sweet berry honeysuckle. Berries of blue honeysuckle are a rich source of phenolic compounds, which according to many authors, account for up to 4% of fresh weight [16]. They reveal especially high content of anthocyanins, with cyanidin, pelargonidin, and peonidin derivatives, which exhibit high contribution to fruit color. Additionally, these berries contain various phenolic acids, flavonols, flavones, and flavan-3-ols [6,13,17,18]. They appear to be also a good source of iridoid compounds with loganic acid being the predominant one [14,19,20,21].

In many studies, it was confirmed that all these substances exhibit wide range of biological activities, like chemoprotective, antimicrobial, anti-inflammatory, antiviral, and anticancer [4,16,20,22,23,24,25]. Due to this fact, there is a high interest in the application of these berries not only for consumption as fresh fruits but also in processed products. Their extracts are richer in bioactive compounds comparing to raw material and therefore may be preferred in functional food production [4,26,27].

Hydrocolloids are the most often applied in yoghurt manufacturing to improve its characteristics by stabilizing its body and texture and for syneresis reduction [28]. The effects of different such substances on yoghurt rheological properties were widely evaluated [29,30,31,32,33]. Among hydrocolloids, starch appears to be an attractive ingredient due to its wide accessibility, low price, and functional properties. Various physical, chemical, and enzymatic modifications were also used to improve its properties and facilitate its utilization for different purposes [34]. Among such modified products, special attention is paid to resistant starch, which in recent years has attracted much attention because of its health profits. This starch is indigestible in the upper parts of the human gastrointestinal tract. After ingestion, at the end of the digestive system, fecal microflora ferments it to short chain fatty acids characterized by different physiological and probiotic effects [35,36]. There are four different types of starch, which differ in their physical and chemical characteristics [37].

Numerous food products have been enriched with resistant starch, mainly baked products, pasta or battered foods [38]. It was also used in the dairy industry for fat substitution in low fat or imitation cheese products and yoghurt [31,39,40]. The new preparation of resistant retrograded and acetylated starch, obtained by Kapelko et al. [41] is characterized by higher water solubility and swelling power and lower susceptibility to amyloglucosidase in comparison to the non-acetylated preparations. Therefore, it may be considered as a functional ingredient in yoghurt. Such modified starch preparation and honeysuckle berries polyphenolic dry extract were used in the undertaken studies to evaluate their potential acceptability as functional additives in yoghurt production, which increase properties and healthy effects of this product.

## 2. Material and Methods

### 2.1. Reagents

All chemicals used were of analytical and HPLC grades and were purchased from Sigma Aldrich (Steinheim, Germany). Cyanidin 3-*O*-glucoside (Cy glc), loganic acid (LA), and loganin (L) were provided by Extrasynthese (Lyon Nord, France). MRS and M-17 agar were obtained from Merck (Kenilworth, NJ, USA).

### 2.2. Extraction of Phenolic Compounds from Honeysuckle Berries

The extraction in details was described by Szołtysik et al. [42].

### 2.3. Preparation of Resistant Starch

Preparation was made according to the method described by Kapelko et al. [41]. Retrograded and acetylated starch with degree of substitution 0.11 was used for the research. Retrogradation was performed by freezing and then defrosting 4% gel; for the acetylation of 100 g starch, 13 mL acetic anhydride was used.

### 2.4. The Total Phenolic Compounds (TPC) Content Determination

The determination of total phenolic compounds (TPC) was analyzed as described by Singleton [43]. Gallic acid was used as a standard. TPC amount was expressed as mg gallic acid equivalent (GAE) per 100 g of fresh weight (FW).

### 2.5. Antioxidant Activity

Antioxidant activities were analyzed using three tests. The DPPH radical scavenging activity was determined according to the method of Yen and Chen [44]. The ABTS method as described by Re et al. (1999) [45], while ferric reducing antioxidant power (FRAP) was measured according to Benzie and Strain [46]. All antioxidant activities determinations were expressed in mmol Trolox equivalent (TE) per 100 g in case of fruit and purified extract or per 1 L in case of yoghurts. The calibration curve in the range of 0.01–5.00 mmol of Trolox was used for the quantification of these activities.

### 2.6. Yoghurt Preparation

Yogurt was made from pasteurized 3.2% fat milk. The milk dry matter content before heat treatment (90 °C, 10 min.) was increased by 2% with skim milk powder (SMP). Its chemical composition was: total solids 13.6%, protein 3.9%, fat 3.22% and carbohydrate 5.74%. Experimental yogurts were additionally enriched with honeysuckle dry extract and resistant starch added individually at concentration 0.1% (samples 0.1F and 0.1S) and in mixture at concentration of each of them 0.1% (0.1F+S) and 0.2% (0.2F+S). The control yogurt was made only with the SMP. Starch preparation (1g) was added to 25 mL of milk and heated to 90 °C; after gelatinization, it was cooled to ca. 40 °C and added to milk for yoghurt production. Dry preparation of honeysuckle berry was dissolved in milk. Yoghurt culture (CHR Hansen) was added to milk at concentration of 2%. Incubation was carried out at the temperature of 43–45 °C until a pH of 4.6–4.7 was reached (about 4,5 h). Yoghurt variants were analyzed in the 1st day after production and 7 and 14 days of storage at 4 °C. Three cups of each yogurt variants were used for physicochemical, microbial and antioxidant activity determinations.

### 2.7. Acidity Analysis of Yoghurt

The pH of the samples was measured by using InoLab pH-meter. Titratable acidity was determined according to the Soxhlet–Henkel method and expressed in grams of lactic acid per liter.

### 2.8. Color Measurement

The surface color of yogurt samples was measured by Minolta Chroma Meter CR-400 (Konica Minolta, Japan) and expressed in the L/(lightness; 100 = white, 0 = black), a/(redness; ±, red; green) and b/(yellowness; ±, yellow; blue) values. Calibration readings of the reference were carried out using a white plate.

### 2.9. Rheological Properties

Yogurt samples were equilibrated at room temperature for 30 min. prior to rheology analysis. Their assessment was carried out using a Haake Rheo Stress 6000 rotational rheometer with a Haake A10 thermostatic bath and UTM Controller (Thermo Electron GmbH, Langenselbold, Germany).

The measurement was performed at a constant temperature (20 °C) using a cone/plate (cone C60/1° Ti L No. 222-1868/stainless steel plate TMP60 No. 222-1891) geometry system with a gap of 1 mm for all samples. At each measurement, 1mL yogurt sample was applied to the surface of the plate. The viscosity [Pa s] was determined three times for each sample with ramp shear rate in the range of 0 to 1000 s^−1^ over 3 min. Apparent viscosity was analyzed at a shear rate of 100 s^−1^.

### 2.10. Microbiological Analysis

Starter bacteria enumeration was carried out by using selected media. *Streptococcus thermophilus* was counted on M-17 and *Lactobacillus delbrueckii subsp. bulgaricus* on MRS agar (pH 5.4), according to ISO 7889/IDF 117 as described by Szołtysik et al. [42].

### 2.11. Whey Separation and Free Amino Groups Content Determination

Yoghurt sample was centrifuged in SIGMA 3K15 centrifuge at 5200 g for 15 min at temp. 40 °C, and the obtained whey was filtered by a 0.45 µm syringe filter (Millex GP, Merck Milipore LTD, Darmstadt, Germany). The concentration of free amino groups (expressed as μM Glycine/100 mL), after relevant whey dilution in 0.05 M borax-HCl, pH 8.2, was determined using trinitrobenzene sulfonic acid (TNBS, Sigma, St. Louis, MO, USA) according to Kuchroo [47].

### 2.12. Extraction and Fractionation of Phenolic Compounds from Yoghurt

Extraction of phenolic compounds from yoghurt was conducted with acidified methanol as described by Trigueros et al. [48]. The HPLC-PDA method, previously described by Kucharska et al. [20] was used for their fractionation. Anthocyanins were expressed as cyanidin 3-*O*-glucoside, Loganic acid and derivatives were expressed as lognic acid and loganin and derivatives as loganin. The results were expressed as mg per 1 L.

### 2.13. Statistical Analyses

Graphs, mean values (X) and standard deviation (SD) were made in excel spreadsheet (Microsoft Office version 2016). Data were analyzed by one-way ANOVA with Statistica 13.1 software (TIBCO, Palo Alto, CA, USA). Differences between means were determined by Duncan′s test (*p* < 0.05). All experiments were carried out in triplicate.

## 3. Results and Discussion

In the undertaken research, two functional additives were used in yoghurt production: the extract of honeysuckle berries and resistant starch, which were applied to milk individually and as mixtures in the amount of 0.1 and 0.2%.

The fruit preparation, obtained with the yield of 1.6%, was characterized by the high total polyphenols amount (TPC) 31.10 g expressed as gallic acid equivalents (GAE) 100 g^−1^ FW. High level of TPC in honeysuckle berries was also reported by other authors [6,19,49]. According to Rupasighe et al. [49] their content determined by the Folin–Ciocalteu assay ranged from 634 to 1154 mg GAE 100 g^−1^ FW, while acc. to Rop et al. [12] it was from 575 to 903 mg GAE 100 g^−1^ FW.

The preparation exhibited high antioxidant potential, determined in ABTS, DPPH and FRAP tests, at the levels of 372.41, 296.63 and 284.39 mmol TE/100 g, respectively (Table 1).

In comparison to other plant extracts, it was characterized by much higher concentration of polyphenolic compounds and antioxidant activity [50,51,52,53]. Several studies have revealed that phenolic compounds in fruits or other parts of plants are associated with their antioxidant activities. They may act as reducing agents, hydrogen donors, and singlet oxygen quenchers and also may have potential of chelating metal ions [54,55].

**Figure 1:**
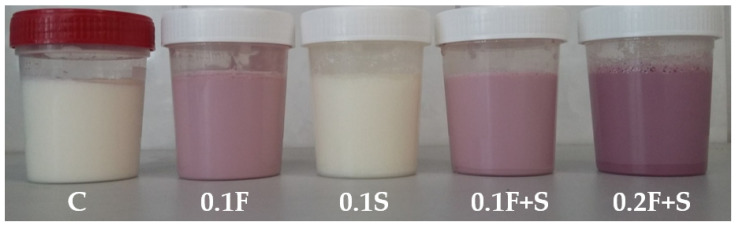
Color of yoghurt samples with different amounts of honeysuckle berries extract and resistant starch preparation after 14 days of storage. C: control, 0.1F: 0.1% addition of the honeysuckle berries extract; 0.1S: 0.1% addition of resistant starch preparation; 0.1F+S: 0.1% addition of the honeysuckle berries extract and resistant starch preparation (each of the additives); 0.2F+S: 0.2% addition of the honeysuckle berries extract and resistant starch preparation (each of the additives).

Fruits of honeysuckle barriers are known as a rich source of this kind of compounds anthocyanins as well as iridoids [20,21]. Introduction of the fruit extract to yoghurts, individually as well as in a mixture with modified starch preparation, gave yoghurts the intensive purple color, as shown in Figure 1.

Color of food products is a very important quality parameter which affects consumer’s acceptability. Introduction of fruits to yoghurts, which changed their color and taste, has led to the increase of marketability and consumption of these products [56]. Fruits extracts, rich in anthocyanins, which have also an array of health-promoting benefits, are especially attractive food coloring additives [57,58,59].

Analyzing the results of yoghurts color determination in L* a* b* system (Table 2) high diversity of the parameter was noticed (*p* < 0.05) depending on the used additive. The lightness parameter L* was the highest for control sample and yoghurt with 0.1% starch preparation as compared to the samples fortified with honeysuckle berry extract, in which values of this parameter decreased as the concentration of the extract was elevated.

In control yoghurt and yoghurt with 0.1% starch preparation, the redness factor a* was negative, while in samples enriched with berry extract took positive values. Different observations were made in these samples for values of yellowness factor b*, which were negative only in yoghurts containing berry extract. Yogurts with higher levels of honeysuckle berry extract showed more blueness. During storage period, all color parameters of control yoghurt and with addition of 0.1% of resistant retrograded starch were at similar level and did not undergo much change.

High color similarity was observed in yoghurts with addition of 0.1% of fruit preparations alone as well as with combination of hydrocolloid additive. In those yoghurts and in yoghurts with addition of 0.2% of both additives, significant changes were observed during yoghurt storage. More apparent changes were observed in sample with addition of 0.2% of both additives. The values of L* parameter increased, while the values of a* and b* decreased, which may point to low stability of anthocyanins of honeysuckle berries. Similar yoghurt color changes were observed also by Nguyen and Hwang [32]. Moreover, according to Ścibisz et al. [7] color changes in food products may be a result of lower stability of anthocyanins present in added fruit extract.

The changes of pH and titratable acidity of yogurts during the storage period are presented in Table 3. After one day of storage, pH value of yoghurts was the highest, it was 4.51 in control and in yoghurt with 0.1% of starch preparation (0.1S) while in other samples ranged between 4.53–4.58. During two weeks, the pH of all yogurt samples decreased to the lowest level of 4.35 and 4.45 in control yoghurt and in yoghurt enriched with 0.2% of both additives (0.2F+S), respectively. With pH decrease during yoghurts storage, in parallel, a significant (*p* < 0.05) increase of titratable acidity was observed. It reached the highest value of 1.19% of lactic acid in yoghurt fortified with 0.2% of two additives, whereas in control, it was lower by 0.09%. The increase of acidity of yoghurt and drop of its pH resulted from metabolic activity of lactic acid bacteria producing lactic acid. Similar tendencies in acidity changes during yoghurts’ cool storage were pointed out by other authors, who used dry plant preparation (wine grape pomace powder, papaya peel powder) as additives to yoghurt [60,61].

Another important feature of yoghurt quality is its texture. The parameter is influenced by the gelation of milk proteins during the fermentation process conducted by thermophilic lactic acid bacteria and also by the many other factors of technological process i.e., solids content of milk, its heat treatment, type and quantity of starter culture and storage conditions of the final product. Many authors analyzed yoghurts texture parameters depending on different additives used [62,63,64]. Especially beneficial are additives belonging to the group of hydrocolloids, such as starch of different origin and its modified types, which elevate the dry matter content and prevent syneresis [28].

In the analyzed yoghurts, the rheological properties were evaluated by the viscosity analysis. The analysis (Table 4) showed a statistically significant (*p* < 0.05) influence of the recipe as well as the time of products storage on their viscosity. In comparison to control, the increase of viscosity was noted in yoghurts enriched in starch preparation alone and in combination with polyphenols. The parameter reached the highest values 0.428 ± 0.029 Pa s in samples with 0.2% addition of both substances. While in samples with addition of 0.1% of honeysuckle barriers alone (0.1F), no significant differences (*p* > 0.05) in viscosity were noted comparing to control; however, during storage of all yoghurt variants, the parameter was lowering. Mwizerwa et al. [31], who investigated rheological parameters of yoghurts enriched at different levels (0.1%, 0.5% and 1%) with cassava starch rich in resistant starch, observed an increase of their viscosity with higher addition of resistant starch, but during cold storage, it decreased, especially in yoghurts with higher concentration of additive. Nguyen et al. [65] also investigated the effect of different hydrocolloids on texture, rheology and tribology of low-fat yoghurts and showed that relatively higher level of modified starch is required to increase their firmness.

Many authors showed that addition of polyphenolic preparations to yoghurt causes the decrease of their viscosity [66]. El-Said et al. [54] observed the decreasing viscosity of the stirred yoghurt with elevation of the added pomegranate peels extract. This effect might be related to the formation of milk proteins and polyphenols complexes, which may induce protein unfolding and the creation of insoluble complexes [9,67,68]. Furthermore, the complex formation appeared to be affected by the phenols concentration and in turn determines the extent of the aggregation phenomena. The extent of this aggregation may be also the reason of different susceptibility of milk proteins to enzymatic hydrolysis by lactic acid bacteria. Ni et al. [10], in their study, showed lower degree of proteins hydrolysis and release of peptides of antidiabetic activity in yoghurts fortified with aqueous extracts from salal berry of higher polyphenols concentration than in samples produced with extract from blackcurrant pomace poorer in these compounds.

In our research, we determined the free amino group content (FAG) which reflects the hydrolytic changes of milk proteins due to the presence of metabolically active starter culture (Table 5). On the first day after production, the contents of FAG were noticeably higher (*p* < 0.05) ca. by 10 μMGly/100mL in yoghurts fortified with modified starch added individually or in combination with polyphenolic preparation at concentration of 0.2% (i.e., 108.7 ± 0.64 and 110.65 ± 1.01 μMGly/100 mL, respectively) in comparison to control and samples with 0.1% addition of polyphenolic preparation. During storage time, the differences in FAG content among analyzed samples persisted. However, in all samples, gradual increase of FAG was observed. It reached the highest level (128 128 μM gly/100 mL) in yoghurt with 0.2% addition of both ingredients (0.2F+S).

Sah et al. [69] while using the addition of pineapple waste powders (dried pineapple peel and pomace powder) rich in dietary fiber and essential minerals showed their stimulating effect on the growth of lactic acid bacteria (LAB), especially probiotic strains. It resulted also in increase of proteolysis in yoghurt and in generation of peptides with antioxidant activity.

The differences in the starter microflora count are presented in Table 6. The highest count of both analyzed species, *S. thermophilus* and *Lb. bulgaricus,* were determined in all samples on the first day after production. In control samples as well as in those with 0.1% addition of polyphenolic preparation, the count was at a similar level during 14 days of storage.

In yoghurts enriched with modified starch alone and in combination with 0.1 and 0.2% polyphenolic preparation during the whole storage, time was higher and statistically significant (*p* < 0,05). On the first day of production, the count of *S thermophilus* was determined at the level of 8.94–9.04 log10 cfu/g, while the number of *Lb. bulgaricus* was lower 8.08–8.22 log10 cfu/g. Our results are similar to the data published by Ni et al. [62], who observed in yoghurts fortified with fruits extracts higher viable counts for *S. thermophilus* compared with *Lb. bulgaricus*, probably due to the documented oxygen sensitivity of *Lb. bulgaricus*.

During yoghurt storage, the decrease of both species count was observed, but still, after 14 days of storage, the number was above the minimal level of >7 log CFU/mL for viable counts in fermented dairy products indicated in Codex Alimentarius [70].

It is noteworthy that the count of both species was the highest in yoghurts enriched with 0.2% of both additives. In those yoghurts, also the highest FAG content was determined. The obtained results showed the stimulating effect, especially of starch preparation on the growth of starter bacteria. However, citing Abdel-Hamid et al. [71], other authors, who used different yoghurt additives such as rice bran, banana fiber or pineapple waste powder, did not observe any influence of those preparations on the growth of yoghurt microflora.

In our research, the honeysuckle berries preparation alone showed no significant effect on starter culture count. Similarly, Chouchouli et al. [72] showed that application of grape seed extracts to yoghurts did not cause significant change in the populations of lactic acid bacteria compared to the control; however, their total number decreased during storage, and this effect was more noticeable during storage longer than 14 days. In contrary Nguyen and Hwang [32], who applied to yoghurt the 2% and 3% addition of chokeberry juice, observed significantly higher number of analyzed bacteria.

Antioxidant activity of yoghurts, measured with three assays, ABTS, DPPH and FRAP, differed depending on additive used in their production (Table 7). In control sample and yogurt with 0.1% of retrograded acetylated starch, it was the lowest and did not change significantly during 14 days of cold storage. Only incorporation of honeysuckle berry preparation into yogurt resulted in higher antioxidant activity, expressed by ABTS method, which increased compared to control (0.10 ± 0.06 µmol TE/L) in samples with 0.1 and 0.2% of additive ca. 7 and 10 times, respectively. With the use of FRAP test, this increase was even higher (ca. 8 and 13 times, respectively), while with DPPH method, it was ca. 4 and 6, respectively. Results showed that antioxidant activity was positively correlated with the dose of applied preparation. The highest values were determined in samples with 0.2% of its addition. It is worthy to notice that in yoghurts enriched only with 0.1% of berry extract, independently of evaluation test used, it was always higher than in yoghurt supplemented with both additives, which may suggest binding of polyphenols by starch. Similar interactions of phenolic compounds and starch were discussed by Zhu et al. [73], who concluded that these compounds interact to form inclusion complex formed by amylose single helices facilitated by hydrophobic effect or complex with much weaker binding through hydrogen bonds. Our results are in line with those described by Ramos et al. [74], who used herbal polyphenol extract alone and in combination with sweet potato pulp rich in dietary fiber to ferment milks. Authors showed that additives affected the texture profile of the products and increased their antioxidant activity. This activity measured by FRAP method was higher in fermented milks with phenolic extract alone than in samples manufactured with both additives, which according to authors might be related to better solubilization of reducing substances in these products than in samples containing also sweet potato, which would have hindered the solubilization of antioxidants.

During the storage period, antioxidant activities in all yoghurt samples containing berry extract showed, in the first week, an increasing tendency, but in the second week, they were lowering. However, the observed differences appeared statistically insignificant (*p* > 0.05). Results obtained in our studies confirmed good stability of antioxidant activities in yoghurts with addition of honeysuckle berries extract, which may be also a result of the milk bioactive compounds presence, especially peptides, released by microbial proteases from milk proteins, which also function as antioxidants.

Good stability of antioxidant activity in yoghurt was observed also by other authors. Dabija et al. [66], determining antioxidant stability of yoghurts enriched with herbs extracts, noticed that it was increasing during storage. Moreover, Zhang et al. [75], using DPPH and ABTS assays, showed the stability of radical-scavenging activity in yoghurts with addition of moringa leaf extract during their storage. Raikos et al. [11], evaluating yoghurt beverages fortified with salal berries and blackcurrant pomace extracts, showed that antioxidant capacity of products was maintained during cold storage.

Many authors evaluating yoghurts fortified with extracts from different plants sources discovered that their antioxidant activity was positively correlated with the amount of anthocyanins; however, those compounds are characterized by quick degradation and instability [20,76]. Therefore, they often observed their significant reduction in products during storage, but the dynamics of the process was dependent on the source of the phenols, which was shown in the studies by Ścibisz et al. [7].

The honeysuckle berries are characterized by the high content of polyphenolic compounds. Kucharska et al. [20] in their research determined among polyphenols, 35 compounds, including flavonols, flavanonols, flavones, flavan-3-ols, phenolic acids and anthocyanins. The last ones were predominat. These authors determined also 15 iridoids compounds, including loganic acid, loganin, sweroside, their derivatives and epimeric pairs of loganic acid and loganin. The contents of anthocyanins and iridoids, as they showed, in different cultivars and genotypes of honeysuckle berries, were between 150.04 mg/100 g FW and 653.95 mg/100 g FW and between 128.42 mg/100 g FW and 372 mg/100 g FW, respectively.

In yoghurts supplemented with honeysuckle berries preparation, both of the mentioned groups of compounds were detected (Table 8 and Table 9). Anthocyanins were represented by 6 compounds, among which 3-*O*-glucoside was predominant, similar as in berries [20]. Guimaraes et al. [77] reported that this compound was also major anthocyanin in *Rosa canina* fruits. In the analyzed samples, the content of this compound accounted for 88–89% of all anthocyanins. Its concentration in samples 0.1F, 0.1F+S and 0.2F+S the next day after production was 48.63 ± 0.74, 41.21 ± 0.27 and 85.32 ± 0.14, respectively. Other compounds from this group present in yoghurts were cyanidin 3-*O*-rutinoside, peonidin 3-*O*-glucoside, cyanidin 3,5-*O*-diglucoside, peonidin 3-*O*-rutinoside and peonidin 3,5-*O*-diglucoside.

The concentration of iridoids in yoghurts was approximately two times higher in comparison to anthocyanins (Table 9). One day after production, their contents in samples 0.1F, 0.1F+S and 0.2F+S were 105.73 mg/L 96.66 mg/L and 188.56 mg/L, respectively. The main compounds were sweroside and loganin comprising between 40–46% of the total quantity of iridoids, followed by loganin 7-*O*-pentoside, 7-epi-loganic acid 7-*O*-pentoside, loganic acid 7-*O*-pentoside and loganic acid, which revealed the lowest concentration, contrary to its concentration in fruits, where it was dominant compound as was showed by Kucharska et al. [20].

In general, the level of both anthocyanins and iridoids in yoghurts was related to the dose of the honeysuckle berries preparation. The highest content was determined in yoghurts enriched with 0.2% of both additives, the lowest in samples with 0.1% addition of the preparation. In yoghurts with 0.1% addition of the berries preparation alone, their concentration was always higher than in the same samples containing also modified starch. The obtained results confirm the possible interaction between honeysuckle berries compounds and polysaccharide.

During yoghurt storage, the statistically significant (*p* < 0.05) decrease of both groups of analyzed compounds content was observed. It was higher for anthocyanins than for iridoids. In samples 0.1F, 0.1F+S and 0.2F+S, the content of anthocyanins lowered after 14 day of storage from the values of 54.89mg/L, 46.55 mg/L and 95.76 mg/L to 31.66mg/L (42.33% decrease), 27.29mg/L (41.38% decrease) and 59.98mg/L (37.37% decrease), respectively.

Despite the significant decrease of anthocyanins content in yoghurts, the antioxidant activity remained stable. It is possible that also higher content of iridoids could complement antioxidant properties of phenolic compounds. Degradation of anthocyanins in fruit yogurt products was observed by many authors in grape yogurt [78], pomegranate yoghurt [48] and in strawberry yogurt [9].

The content of iridoids in analyzed yoghurts during their storage was also decreasing, however to a lesser extent in comparison to anthocyanins. After 14 days of storage, their content decreased in about 32–37%. Iridoids, due to numerous desirable bioactivities such as anti-inflammatory, antimicrobial, immunomodulatory, neuroprotective and antitumor activities, appeared to be especially attractive food additives [4,20]. As opposed to polyphenols, these compounds are rarely found in fruits [20]. One of their sources is honeysuckle berries, rich also in anthocyanins. Therefore, preparation obtained from these berries comprising both groups of compounds seems to be a promising additive in the production of functional food, like yoghurt.

## 4. Conclusions

The preparation from honeysuckle berries, rich in polyphenols and iridoid compounds, and resistant starch, characterized by good solubility in water, high swelling power and low susceptibility to amyloglucosidase, may be an attractive additive for functional food production. The first one, applied in yoghurts production, resulted in an increase of their antioxidant potential and gave them a pleasant deep purple color. The second one revealed an especially stimulating effect on starter bacteria growth and caused an increase in the proteolytic degradation in yoghurt. Both additives added to yoghurts individually and in combination did not cause radical changes of their physicochemical properties, as acidity and rheological parameters. Although anthocyanins and iridoids lowered during two weeks of yoghurts cold storage (at higher rate in the case of anthocyanins), their free radical scavenging activity and ferric reducing antioxidant power remained stable.

## Figures and Tables

**Table 1:** Total polyphenols and antioxidant activity of honeysuckle extract.

Parameter	Content
Total Polyphenols	31.10 ± 0.88 g GAE/100 g
DPPH	296.63 ± 5.68 mmol TE/100 g
ABTS	372.41 ± 7.13 mmol TE/100 g
FRAP	284.39 ± 11.79 mmol TE/100 g

**Table 2:** Changes in color parameters of yoghurts during storage.

Storage Time (Days)	Control	0.1F	0.1S	0.1F+S	0.2F+S
	L*				
1	90.25 ± 0.22 ^f^	61.28 ± 0.13 ^c^	89.79 ± 0.39 ^f^	61.3 ± 0.33 ^c^	53.17 ± 0.41 ^a^
7	90.06 ± 0.15 ^f^	62.17 ± 0.33 ^d^	89.91 ± 0.19 ^f^	61.88 ± 0.42 ^d^	53.22 ± 0.16 ^a^
14	89.91 ± 0.37 ^f^	63.3 ± 0.5 ^e^	90.06 ± 0.58 ^f^	63.78 ± 0.33 ^e^	60.69 ± 0.09 ^b^
a*					
1	−3.61 ± 0.13 ^a^	13.79 ± 0.11 ^bc^	−3.38 ± 0.11 ^a^	13.9 ± 0.12 ^c^	17.55 ± 0.14 ^d^
7	−3.5 ± 0.12 ^a^	13.76 ± 0.12 ^bc^	−3.38 ± 0.05 ^a^	13.58 ± 0.11 ^b^	17.6 ± 0.14 ^d^
14	−3.51 ± 0.11 ^a^	13.71 ± 0.2 ^bc^	−3.37 ± 0.04 ^a^	13.66 ± 0.31 ^bc^	19.06 ± 0.05 ^e^
b*					
1	7.32 ± 0.2 ^e^	−1.23 ± 0.03 ^c^	7.31 ± 0.12 ^e^	−1.26 ± 0.03 ^c^	−1.73 ± 0.01 ^a^
7	7.22 ± 0.1 ^e^	−1.21 ± 0.02 ^c^	7.37 ± 0.06 ^e^	−1.25 ± 0.04 ^c^	−1.54 ± 0.05 ^b^
14	7.3 ± 0.09 ^e^	−0.8 ± 0.07 ^d^	7.34 ± 0.07 ^e^	−0.73 ± 0.24 ^d^	−1.32 ± 0.04 ^c^

C: control, 0.1F: 0.1% addition of the honeysuckle berries extract; 0.1S: 0.1% addition of resistant starch preparation; 0.1F+S: 0.1% addition of the honeysuckle berries extract and resistant starch preparation (each of the additives); 0.2F+S: 0.2% addition of the honeysuckle berries extract and resistant starch preparation (each of the additives).Values X ± SD (*n* = 3); mean values with different letters (^a^, ^b^, ^c^, etc.) within a single color component are statistically different (*p* < 0.05).

**Table 3:** Changes of pH and total acidity (%) in yoghurt samples during storage.

Storage Time (Days)	Control	0.1F	0.1S	0.1F+S	0.2F+S
	pH				
1	4.51 ± 0.06 ^cdef^	4.55 ± 0.07 ^ef^	4.51 ± 0.01 ^cdef^	4.53 ± 0.04 ^def^	4.58 ± 0.06 ^f^
7	4.49 ± 0.13 ^bcdef^	4.45 ± 0.04 ^abcde^	4.47 ± 0.03 ^bcdef^	4.45 ± 0.07 ^abcde^	4.55 ± 0.07 ^ef^
14	4.35 ± 0.08 ^a^	4.42 ± 0.02 ^abcd^	4.4 ± 0.03 ^abc^	4.38 ± 0.06 ^ab^	4.45 ± 0.04 ^abcde^
**Total Acidity [%]**					
1	0.98 ± 0.01 ^a^	1.09 ± 0.02 ^cde^	1.07 ± 0.02 ^bc^	1.08 ± 0.03 ^bcd^	1.08 ± 0.02 ^bcd^
7	1.03 ± 0.02 ^b^	1.11 ± 0.01 ^cde^	1.12 ± 0.05 ^cdef^	1.12 ± 0.04 ^cdef^	1.14 ± 0.02 ^efg^
14	1.1 ± 0.02 ^cde^	1.17 ± 0.06 ^fg^	1.13 ± 0.02 ^def^	1.14 ± 0.02 ^efg^	1.19 ± 0.01 ^g^

C: control, 0.1F: 0.1% addition of the honeysuckle berries extract; 0.1S: 0.1% addition of resistant starch preparation; 0.1F+S: 0.1% addition of the honeysuckle berries extract and resistant starch preparation (each of the additives); 0.2F+S: 0.2% addition of the honeysuckle berries extract and resistant starch preparation (each of the additives). Values X ± SD (*n* = 3); mean values with different letters (^a^, ^b^, ^c^, etc.) within the experimental yoghurt variant are statistically different (*p* < 0.05).

**Table 4:** Viscosity changes of yoghurt samples during storage.

Storage Time (Days)	Control	0.1F	0.1S	0.1F+S	0.2F+S
	Viscosity (Pa s)				
1	0.293 ± 0.042 ^bcde^	0.287 ± 0.021 ^bcde^	0.317 ± 0.026 ^de^	0.333 ± 0.025 ^ef^	0.428 ± 0.029 ^g^
7	0.260 ± 0.026 ^abcd^	0.237 ± 0.021 ^ab^	0.280 ± 0.062 ^bcde^	0.303 ± 0.038 ^cde^	0.383 ± 0.035 ^fg^
14	0.253 ± 0.032 ^abcd^	0.21 ± 0.026 ^a^	0.243 ± 0.05 ^abc^	0.257 ± 0.015 ^abcd^	0.339 ± 0.023 ^ef^

C: control, 0.1F: 0.1% addition of the honeysuckle berries extract; 0.1S: 0.1% addition of resistant starch preparation; 0.1F+S: 0.1% addition of the honeysuckle berries extract and resistant starch preparation (each of the additives); 0.2F+S: 0.2% addition of the honeysuckle berries extract and resistant starch preparation (each of the additives). Values X ± SD (*n* = 3); mean values with different letters (^a^, ^b^, ^c^, etc.) are statistically different within the experimental yoghurt variant (*p* < 0.05). Apparent viscosity was determined at a shear rate of 100 s^−1^.

**Table 5:** Free amino groups content in yoghurt (μmol Gly/100 g) during storage.

Storage Time (Days)	Control	0.1F	0.1S	0.1F+S	0.2F+S
	μMGly/100 g				
1	98.05 ± 0.51 ^a^	97.98 ± 0.22 ^a^	108.7 ± 0.64 ^c^	98.51 ± 0.14 ^a^	110.65 ± 1.01 ^d^
7	106.53 ± 0.36 ^b^	107.09 ± 0.25 ^b^	121.41 ± 0.22 ^f^	117.86 ± 0.22 ^e^	123.03 ± 0.31 ^g^
14	111.07 ± 1.63 ^d^	111.38 ± 1.14 ^d^	127.19 ± 0.3 ^i^	124.82 ± 0.5 ^h^	128.26 ± 0.37 ^i^

C: control, 0.1F: 0.1% addition of the honeysuckle berries extract; 0.1S: 0.1% addition of resistant starch preparation; 0.1F+S: 0.1% addition of the honeysuckle berries extract and resistant starch preparation (each of the additives); 0.2F+S: 0.2% addition of the honeysuckle berries extract and resistant starch preparation (each of the additives). Values X ± SD (*n* = 3); mean values with different letters (^a^, ^b^, ^c^, etc.) within the experimental yoghurt variant are statistically different (*p* < 0.05).

**Table 6:** Count of *Lb. bulgaricus* and *S. thermophilus* in yoghurt during storage (log CFU/mL).

Storage Time (Days)	Control	0.1F	0.1S	0.1F+S	0.2F+S
	*Lactobacillus bulgaricus*				
1	7.75 ± 0.07 ^de^	7.85 ± 0.07 ^ef^	8.21 ± 0.08 ^h^	8.08 ± 0.06 ^g^	8.22 ± 0.09 ^h^
7	7.62 ± 0.03 ^c^	7.65 ± 0.06 ^cd^	8.07 ± 0.05 ^g^	7.9 ± 0.01 ^f^	8.14 ± 0.06 ^gh^
14	7.3 ± 0.08 ^a^	7.51 ± 0.02 ^b^	7.89 ± 0.05 ^f^	7.84 ± 0.07 ^ef^	7.92 ± 0.09 ^f^
***Streptococcus thermophilus***					
1	8.51 ± 0.14 ^abcd^	8.54 ± 0.42 ^abcd^	8.95 ± 0.54 ^cde^	8.94 ± 0.22 ^cde^	9.04 ± 0.22 ^e^
7	8.49 ± 0.02 ^abc^	8.48 ± 0.28 ^abc^	8.92 ± 0.11 ^cde^	9 ± 0.33 ^de^	8.99 ± 0.07 ^de^
14	8.34 ± 0.16 ^ab^	8.3 ± 0.28 ^a^	8.76 ± 0.2 ^abcde^	8.76 ± 0.11 ^abcde^	8.83 ± 0.21 ^bcde^

C: control, 0.1F: 0.1% addition of the honeysuckle berries extract; 0.1S: 0.1% addition of resistant starch preparation; 0.1F+S: 0.1% addition of the honeysuckle berries extract and resistant starch preparation (each of the additives); 0.2F+S: 0.2% addition of the honeysuckle berries extract and resistant starch preparation (each of the additives). Values X ± SD (*n* = 3); mean values with different letters (^a^, ^b^, ^c^, etc.) within the experimental yoghurt variant are statistically different (*p* < 0.05).

**Table 7:** Antioxidant activity, measured by ABTS, DPPH and FRAP methods, in yoghurts during storage.

Storage Time (Days)	Control	0.1F	0.1S	0.1F+S	0.2F+S
	ABTS (µmol TE/L)				
1	0.10 ± 0.06 ^a^	0.67 ± 0.07 ^d^	0.11 ± 0.06 ^a^	0.57 ± 0.01 ^b^	1.08 ± 0.04 ^ef^
7	0.13 ± 0.01 ^a^	0.70 ± 0.02 ^d^	0.15 ± 0.01 ^a^	0.70 ± 0.01 ^d^	1.13 ± 0.03 ^f^
14	0.11 ± 0.02 ^a^	0.65 ± 0.04 ^cd^	0.13 ± 0.03 ^a^	0.59 ± 0.04 ^bc^	1.03 ± 0.03 ^e^
**DPPH (µmol TE/L)**					
1	0.10 ± 0.02 ^ab^	0.38 ± 0.01 ^de^	0.08 ± 0.00 ^a^	0.35 ± 0.02 ^cd^	0.58 ± 0.03 ^h^
7	0.12 ± 0.01 ^ab^	0.48 ± 0.03 ^g^	0.13 ± 0.01 ^b^	0.43 ± 0.02 ^f^	0.67 ± 0.03 ^i^
14	0.10 ± 0.02 ^ab^	0.40 ± 0.02 ^ef^	0.13 ± 0.02 ^b^	0.33 ± 0.03 ^c^	0.61 ± 0.04 ^h^
**FRAP (µmol TE/L)**					
1	0.10 ± 0.00 ^a^	0.76 ± 0.02 ^b^	0.12 ± 0.00 ^a^	0.71 ± 0.03 ^b^	1.32 ± 0.2 ^c^
7	0.12 ± 0.01 ^a^	0.83 ± 0.01 ^b^	0.10 ± 0.02 ^a^	0.83 ± 0.02 ^b^	1.35 ± 0.06 ^c^
14	0.13 ± 0.02 ^a^	0.78 ± 0.04 ^b^	0.14 ± 0.06 ^a^	0.73 ± 0.05 ^b^	1.26 ± 0.12 ^c^

C: control, 0.1F: 0.1% addition of the honeysuckle berries extract; 0.1S: 0.1% addition of resistant starch preparation; 0.1F+S: 0.1% addition of the honeysuckle berries extract and resistant starch preparation (each of the additives); 0.2F+S: 0.2% addition of the honeysuckle berries extract and resistant starch preparation (each of the additives). Values X ± SD (*n* = 3); mean values with different letters (^a^, ^b^, ^c^, etc.) within the experimental yoghurt variant are statistically different (*p* < 0.05).

**Table 8:** Changes of anthocyanins contents (mg/L) in yoghurt samples during storage.

Sample	Days	Cy diglc	Pn diglc	Cy glc	Cy rut	Pn glc	Pn rut	Total Anthocyanins
0.1F	1	1.38 ± 0.07 ^c^	0.09 ± 0.03 ^ab^	48.63 ± 0.74 ^f^	2.98 ± 0.06 ^e^	1.54 ± 0.01 ^e^	0.27 ± 0.01 ^b^	54.89 ^f^
	7	1.22 ± 0.06 ^b^	0.07 ± 0.01 ^ab^	37.79 ± 0.28 ^d^	2.58 ± 0.05 ^d^	1.41 ± 0.06 ^d^	0.24 ± 0.02 ^b^	43.31 ^d^
	14	0.95 ± 0.08 ^a^	0.05 ± 0.01 ^a^	28.11 ± 0.62 ^b^	1.63 ± 0.04 ^a^	0.81 ± 0.01 ^a^	0.11 ± 0.03 ^a^	31.66 ^b^
0.1F+S	1	1.17 ± 0.04 ^b^	0.07 ± 0.01 ^ab^	41.21 ± 0.27 ^e^	2.61 ± 0.08 ^d^	1.24 ± 0.06 ^c^	0.25 ± 0.03 ^b^	46.55 ^e^
	7	1.12 ± 0.08 ^b^	0.07 ± 0.01 ^ab^	34.11 ± 0.62 ^c^	2.38 ± 0.04 ^c^	1.01 ± 0.01 ^b^	0.24 ± 0.03 ^b^	38.93 ^c^
	14	0.92 ± 0.09 ^a^	0.05 ± 0.01 ^a^	23.14 ± 0.42 ^a^	2.16 ± 0.07 ^b^	0.80 ± 0.04 ^a^	0.22 ± 0.03 ^b^	27.29 ^a^
0.2F+S	1	2.26 ± 0.06 ^e^	0.13 ± 0.03 ^c^	85.32 ± 0.14 ^i^	5.02 ± 0.04 ^h^	2.51 ± 0.03 ^h^	0.52 ± 0.03 ^e^	95.76 ^i^
	7	2.13 ± 0.06 ^d^	0.11 ± 0.04 ^bc^	65.51 ± 0.13 ^h^	4.03 ± 0.03 ^g^	2.22 ± 0.04 ^g^	0.38 ± 0.02 ^d^	74.38 ^h^
	14	2.03 ± 0.07 ^d^	0.11 ± 0.01 ^bc^	51.95 ± 0.26 ^g^	3.93 ± 0.03 ^f^	1.64 ± 0.03 ^f^	0.33 ± 0.04 ^c^	59.98 ^g^

Cy diglc: cyanidin 3,5-*O*-diglucoside; Pn diglc: peonidin 3,5-*O*-diglucoside; Cy glc: cyanidin 3-*O*-glucoside; Cy rut: cyanidin 3-*O*-rutinoside; Pn glc: peonidin 3-*O*-glucoside; Pn rut: peonidin 3-*O*-rutinosid. C: control, 0.1F: 0.1% addition of the honeysuckle berries extract; 0.1S: 0.1% addition of resistant starch preparation; 0.1F+S: 0.1% addition of the honeysuckle berries extract and resistant starch preparation (each of the additives); 0.2F+S: 0.2% addition of the honeysuckle berries extract and resistant starch preparation (each of the additives). Values are expressed as the mean (*n* = 3) standard deviation. Mean values with different letters (^a^, ^b^, ^c^, etc.) are statistically different (*p* < 0.05).

**Table 9:** Changes of iridoids contents (mg/L) in yoghurt samples during storage.

Sample	Day	LA	LAp	epiLAp	S + L	Lp	Total Iridoids
0.1F	1	7.92 ± 0.27 ^d^	8.49 ± 0.42 ^d^	14.71 ± 0.28 ^d^	49.29 ± 0.21 ^g^	25.33 ± 0.25 ^f^	105.73 ^f^
	7	7.03 ± 0.12 ^c^	8.36 ± 0.19 ^d^	13.52 ± 0.29 ^c^	38.56 ± 0.30 ^d^	20.16 ± 0.16 ^e^	87.63 ^d^
	14	6.34 ± 0.14 ^b^	6.86 ± 0.05 ^b^	12.50 ± 0.44 ^b^	28.18 ± 0.08 ^b^	18.06 ± 0.08 ^b^	71.94 ^b^
0.1F+S	1	10.82 ± 0.48 ^e^	10.70 ± 0.21 ^e^	16.26 ± 0.33 ^e^	39.30 ± 0.16 ^e^	19.59 ± 0.37 ^d^	96.66 ^e^
	7	8.28 ± 0.28 ^d^	7.62 ± 0.26 ^c^	14.96 ± 0.28 ^d^	34.91 ± 0.16 ^c^	18.85 ± 0.11 ^c^	84.62 ^c^
	14	5.32 ± 0.31 ^a^	5.07 ± 0.16 ^a^	11.57 ± 0.18 ^a^	26.95 ± 0.11 ^a^	13.39 ± 0.16 ^a^	60.30 ^a^
0.2F+S	1	16.66 ± 0.29 ^h^	13.23 ± 0.16 ^g^	32.44 ± 0.18 ^h^	82.12 ± 0.11 ^i^	44.12 ± 0.16 ^i^	188.56 ^i^
	7	14.80 ± 0.27 ^g^	12.89 ± 0.18 ^g^	27.22 ± 0.26 ^g^	62.14 ± 0.06 ^h^	37.35 ± 0.09 ^h^	154.40 ^h^
	14	12.03 ± 0.34 ^f^	11.86 ± 0.32 ^f^	21.04 ± 0.15 ^f^	47.05 ± 0.08 ^f^	26.97 ± 0.12 ^g^	118.95 ^g^

Iridoids: LA: loganic acid; Lap: loganic acid 7-*O*-pentoside; epi-Lap: 7-epi-loganic acid 7-*O*-pentoside; S: sweroside; L: loganin; Lp: loganin 7-*O*-pentoside; C: control, 0.1F: 0.1% addition of the honeysuckle berries extract; 0.1S: 0.1% addition of resistant starch preparation; 0.1F+S: 0.1% addition of the honeysuckle berries extract and resistant starch preparation (each of the additives); 0.2F+S: 0.2% addition of the honeysuckle berries extract and resistant starch preparation (each of the additives). Values are expressed as the mean (*n* = 3) standard deviation. Mean values with different letters (^a^, ^b^, ^c^, etc.) are statistically different (*p* < 0.05).
